# A Novel Patient Similarity Network (PSN) Framework Based on Multi-Model Deep Learning for Precision Medicine

**DOI:** 10.3390/jpm12050768

**Published:** 2022-05-10

**Authors:** Alramzana Nujum Navaz, Hadeel T. El-Kassabi, Mohamed Adel Serhani, Abderrahim Oulhaj, Khaled Khalil

**Affiliations:** 1 Department of Information Systems and Security, College of Information Technology, UAE University, Al Ain P.O. Box 15551, United Arab Emirates; 201570182@uaeu.ac.ae; 2 Department of Computer Science and Software Engineering, Concordia University, Montreal, QC H3G 1M8, Canada; hadeel.elkassabi@concordia.ca; 3 Department of Epidemiology and Public Health, College of Medicine and Health Sciences, Khalifa University, Abu Dhabi P.O. Box 17666, United Arab Emirates; abderrahim.oulhaj@ku.ac.ae; 4 Institute of Public Health, College of Medicine and Health Sciences, UAE University, Al Ain P.O. Box 15551, United Arab Emirates; 5 Faculty of Applied Science and Engineering, University of Toronto, Toronto, ON M5S 1A4, Canada; Khaled.khalil@mail.utoronto.ca

**Keywords:** patient, patient similarity network, precision medicine, big data, personalized healthcare, patient-centered framework, deep learning, electronic health records, transformers, BERT, autoencoder

## Abstract

Precision medicine can be defined as the comparison of a new patient with existing patients that have similar characteristics and can be referred to as patient similarity. Several deep learning models have been used to build and apply patient similarity networks (PSNs). However, the challenges related to data heterogeneity and dimensionality make it difficult to use a single model to reduce data dimensionality and capture the features of diverse data types. In this paper, we propose a multi-model PSN that considers heterogeneous static and dynamic data. The combination of deep learning models and PSN allows ample clinical evidence and information extraction against which similar patients can be compared. We use the bidirectional encoder representations from transformers (BERT) to analyze the contextual data and generate word embedding, where semantic features are captured using a convolutional neural network (CNN). Dynamic data are analyzed using a long-short-term-memory (LSTM)-based autoencoder, which reduces data dimensionality and preserves the temporal features of the data. We propose a data fusion approach combining temporal and clinical narrative data to estimate patient similarity. The experiments we conducted proved that our model provides a higher classification accuracy in determining various patient health outcomes when compared with other traditional classification algorithms.

## 1. Introduction

A “one-size-fits-all” approach to medicine is unreliable since some therapies work better in some individuals than others. Precision medicine, which is a recent and innovative approach, considers the individual differences in people’s genes, environmental contexts, and lifestyles. The precision medicine initiative, which was implemented by President Obama in 2015 [1], empowers people to invest and manage their health by providing tailored healthcare. Often, individuals seek examples from other individuals in similar fields to make decisions regarding various life-related matters. For instance, in real life, students make academic and career plans by seeking guidance from their seniors who have taken similar choices and have experienced the same path previously. Physicians take inputs, learn, and adapt based on their previous experience in handling various cases [2]. Similarly, patients seek guidance, recommendations, and medical treatments from patients suffering from similar health conditions. Patient-friendly social websites, such as PatientsLikeMe [3], are platforms on which people with every type of condition share their health experiences, find similar patients, learn how to take control over their health, and participate in their health management. These websites enable information sharing between patients and the provision of advice from healthcare workers. As a result, patient care is improved, and realistic medical research is accelerated. 

Patient similarity analysis [4] aims to classify patients into medically relevant clusters to gain insight into underlying disease mechanisms. Common disease trajectories leading to specific outcomes can be established based on the clustering of patient journeys, which involves all the timeline of medical services and events from admission to discharge/death. This is based on the premise that insights gained using prediction models trained on similar patients’ data are more dependable than those obtained using all available data. The patient similarity network (PSN) model makes it possible for classifiers to be accurate and generalizable. Furthermore, it provides the classifiers with the ability to incorporate heterogeneous data and manage missing information naturally [5]. PSNs are used to handle heterogeneous data by converting each datatype to a similarity network and then easily integrating/aggregating them into one similarity network using, for instance, a fusion algorithm. Moreover, surpasses other classification and clustering algorithms in handling missing data because the existing data can be used in another network if patient data is missing for one network. Additionally, techniques for deep network embedding, graph neural networks, and ordinary neural differential equation models can be implemented using graph analytics algorithms [6]. These approaches are predominantly used in the case of the multimodal patient data associated with the predictive modeling of health hazards and subtyping of diseases. In precision medicine, patient similarity analysis can be used to improve patient outcome prediction and, it is likely to contribute to clinical decision making.

PSN is a new trend that comes under the umbrella of precision medicine, where patients are clustered or classified based on their similarities according to various features. The theory associated with the case similarity of patients can be explained using the following example. If two patients are similar, based on several aspects, their medical case progression is also bound to be similar. Therefore, identifying past patients similar to the current patient could help to provide insights related to disease investigations and potential treatments. Thus, the objective of PSNs is to recommend the appropriate therapy, medicine, and lifestyle changes to the current patient based on relevant data extracted from similar patients, thereby determining the possible clinical outcomes [5].

**Figure 1:**
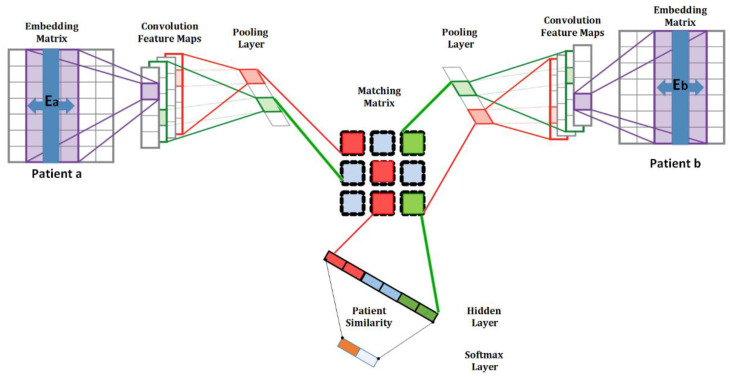
Supervised patient similarity matching framework.

Each input patient data feature is represented as a patient similarity network in this system (PSN) [7]. Each PSN node is an individual patient and an edge between two patients corresponds to pairwise similarity. Using a similarity measure, PSNs can be generated from any available data. Deep learning (DL) based on supervised patient similarity [8], represents patient pairs with embedding matrices (Ea and Eb) passing through convolutional filters and are mapped onto feature maps to train the neural network (Figure 1). Deep embedding patient representations (Pa and Pb) are created for patients by pooling patient feature maps into the intermediate vectors. A symmetrical similarity matrix M with feature vectors is learned to calculate the similarity between patients a and b.

The remainder of this paper is organized as follows. Initially, a comparative study of the existing literature in PSN and the challenges are identified. Further, we propose a hybrid model for PSNs. Then, we present our recommended hybrid model formulation and establish the model using the presented algorithms. Subsequently, we detail our experimental scenarios and discuss the results. Finally, we discuss directions for future work and conclude the paper.

## 2. Related Work

In this section, we present a systematic review of the current literature on PSNs considering the approaches used to build a PSN network, combination PSN models, the PSN application domains in healthcare, and PSN performance evaluation approaches. Further, we identify the challenges associated with the existing studies on PSNs and introduce our approach to solve some of these challenges.

### 2.1. Existing Techniques for Building PSNs

The PSN framework offers reliable, generalizable classifiers that can integrate heterogeneous data and intuitively manage missing information [5]. Table 1 presents some approaches that have been adopted for building patient similarity, including neural networks. DL [9] is an end-to-end deep similarity learning technique that simultaneously learns patient representations and determines the association between the patients using pairwise similarity probability. CNN [10] is applied to investigate the vector representation of raw EHRs and collect important information about patient attributes, and a SoftMax-based supervised classification algorithm is used to discover the similarity between pairs of patients. A recent study [8] proposed a DL architecture (CNN) to evaluate patient similarity based on the temporal matching of patient EHRs represented via medical concept embedding. The similarity between two patients is calculated from the temporal representation, where the records of each patient *p* are represented as a matrix X, with dimension d × N*p*, where d is the dimension and N*p* is the total number of hospital visits for patient *p*.

Generally, two different approaches are adopted to measure patient similarity [24]. In the first approach, patients are clustered using two standard clustering algorithms (K-means and hierarchical clustering) and, in the second, patients are clustered using a supervised technique in which the medication plan is considered as a class variable. An extension of the influence-diagram representation called a similarity network [25] was introduced in the late 1990s, for constructing large and complex influence diagrams. It consists of a similarity graph and a collection of local knowledge maps. The nodes in the similarity graph correspond to the hypotheses and the edges connect similar hypotheses. Similarity networks are an extension of the belief network representation that was the basis of Pathfinder [26], which was a decision-theoretic expert system developed for hematopathology diagnosis.

Intelligent self-learning electronic medical record (ISLEMR) [27] is based on a PSN and considers the principal diagnosis as the similarity assessment input. It can provide treatment plan recommendations and can help in training inexperienced doctors. Patients $P_{i}$ and $\;P_{j}$ have principal diagnoses $D_{i}$ and $D_{j}$, respectively, and the similarity is 1 when $D_{i}$ is the same as $D_{j}$, and when they differ, the similarity is 0. In EASY MODE, patients with the same principal diagnosis as the target patient constitute the most similar patient group. In MIXED MODE similarity, patients’ demographic data, vital signs, and structured lab test results are considered and, in COMPLEX MODE, the dissimilarity matrix is obtained after a series of steps. Initially, all the objects are apart, forming a small cluster by itself. The two most similar objects are combined to form a new cluster and a new dissimilarity matrix is computed. This process is repeated until all objects are reallocated into two clusters.

The patient similarity metric is defined as the cosine of the angle between two patient vectors, called cosine similarity [16]. Clinical temporal data are divided into two types: time series (biosignals) and temporal sequences (time-stamped clinical data). In [13], the authors employed clinical temporal data similarity of workflows to discover cases by comparing the ideal case with particular patient situations. They employed an interval similarity function based on intra-task distance (distance between intervals representing related tasks) and inter-task distance (distance between relations representing comparable tasks). They also considered the possibility of case dissimilarities in tasks that occur in succession.

One instance in which similarity information can be derived from data is when the data ideas are organized hierarchically, and an example of such a concept hierarchy [23] is the World Health Organization’s International Classification of Disease-Version 10 (ICD-10). The semantic meaning of the granular details of severity and complexity of disease conditions, diagnosis, treatment of diseases, etc. are encoded by the ICD-10, and can be considered to measure the supervised distance of patients [7]. Patients can be clustered based on the comparison provided by ICD-10 associations. In the patient–ICD-10 association matrix, each patient is represented as a vector of the associated ICD-10 codes in the space of all the 674 ICD-10 codes. The significance of ICD-10 occurrences was weighed in [28] using the term frequency-inverse document frequency (TF-IDF) measure and cosine similarity to calculate the cosine of the angle between all the vector pairs. Research on phenotype similarity [29] also used TF-IDF to considerably improve the quality of the predicted data.

The American Medical Informatics Association (AMIA) 2019 recommendations, based on a workshop on patient similarity, classify patient similarities into four classes, namely, feature, outcome, exposure, and mixed classes [30]. Each class has unique temporal (snapshot vs. shift over time) characteristics to determine whether it is an entity or an event. Entity means that the characteristics are physical object properties (tumors), whereas events indicate the undertaken procedures. The majority of the PSN methods use a vector-based representation of patients that require the aggregation of medical procedures over a period, resulting in the loss of temporal information. Feature similarity [30] is the capturing of data in a brief span of time known as a “snapshot”. To increase the utility of patient similarity measures, methods to identify variables with the best predictive value for a particular outcome are required. Finding matches in temporal-based endpoints is the focus of outcome similarity [30]. Outcome measurements can be thought of as a “snapshot” of a patient’s health when they are used to match similar patients. Exposure similarity [30] detects patients based on the presence or lack of treatment interventions or other exposures that impact their health condition. Exposure to past lines of therapy is used as an inclusion criterion in clinical studies to improve the accuracy of predicted disease and response to medication. The last major type of similarity is the interplay of these classes, sometimes known as mixed-class similarity [30]. When comorbidity status and treatment exposure are combined in a patient, a mixed measure is created that is more complicated and predictive of genuine patient similarity. In consequence, a “curse of dimensionality” would indicate that no two patients are the same in any meaningful sense, given the nearly endless data required to adequately depict a patient. It is probable that task- and setting-specific computed similarity initiatives will increase its applicability.

Personalized predictive modeling [19] includes patient similarity computation, feature filtering, predictive modeling, and risk factor profiling. A trainable similarity measure called locally supervised metric learning (LSML) that is customizable for a specific disease or condition is used to find patient similarity. According to this study, the usage of static similarity measures, such as Euclidean or Mahalanobis, may not be optimal for all the target disease conditions and proposes a logistic regression (LR) predictive model to compute the risk factor profile, i.e., the risk of diabetes disease onset for the test patient. PSNs within a database management system (NoSQL), in-database data analysis, pre-processing, and patient similarity calculation have been discussed in [31]. DeepPatient [17] leads to more compressed and lower-dimensional representations than the original EHRs, allowing data to scale better using stacked denoising autoencoders. 

Unlike patient similarity, only some studies have been conducted on drug similarity and sequence-based gene-gene similarity. SITAR [32], which is an algorithm for predicting drug targets based on drug-drug and gene–gene similarity computations, performs feature selection and prediction using logistic regression. Semantic similarity metrics can be used to measure phenotypic similarity [33] based on human phenotype ontology to rank diseases. In a federated framework for PSNs across organizations, a privacy-preserving platform [34] was proposed to find similar patients from multiple hospitals without sharing patient-level information. The host genome and reaction, transmission history, and course of the disease will allow us to resolve the current pandemic by initiating precision epidemiology [35], which is a modernized workflow that considers the viral genome. A disease transmission dynamics map has been proposed in [36], using the similarities and dissimilarities of dynamics among many countries rather than patients. 

### 2.2. Combination PSN Models

Other categories of works that consider a combination of PSN models have also been proposed in the literature. CrOss-Modal PseudO-SiamEse network (COMPOSE) [37] is used to address the challenges of patient-trial matching, where the pre-trained BERT is used to generate contextualized word embedding in EHR and semantic features are captured using CNN. The combination model claims 98% accuracy in patient criteria matching. DeepPPPred [38], which is an ensemble classifier employing three versions of deep neural networks (recurrent neural networks (RNN), CNN, and BERT), outperforms its constituent individual neural networks. However, the COMPOSE model is for patient-trial matching and not patient similarity matching, whereas DeepPPPred is for protein classification. The usage of a gated network for clinical semantic textual similarity [39] by fusing the distributed BERT and one-hot representations results in a high Pearson correlation (0.8525), which is greater than those of the baseline system using only BERT by 0.0064 and only one-hot representation by 0.0586. Data fusion using matrix factorization [40] is a data-merging algorithm that can target a specific relation and utilizes the associated, contextual, and system constraint data. This approach claims to model any data that can be represented in a matrix and is used in gene function prediction.

### 2.3. PSN Application in Various Health Domains 

Most of the research studies on patient similarity were rooted in cancer-related domains [18,22,41]. Glioblastoma multiforme (GBM), an aggressive adult brain tumor, was the focus of a previous study [18], in which mRNA expression, DNA methylation, and microRNA (miRNA) expression data were combined. PSN has been employed in identifying hepatocellular carcinoma patients with similar survival times after transarterial chemoembolization (TACE) [22]. EHR indicating diseases, such as diabetes, schizophrenia, and various cancers, was considered in DeepPatient [17]. Personalized predictive models [19] identified the top risk factors for diabetes onset. Patient similarity experiments were conducted on real EHRs for stroke disease collected by the Chinese stroke data center [42], and included data of ischemic heart disease (ICD-10 code: I2) and cerebrovascular disease (ICD-10 code: I6). The multidimensional patient similarity study proposed in [24] used medical comorbidities, laboratory measurements, ejection fraction, vital status, and demographics to identify similar patients for inferring an individual patient’s response to heart failure therapy. Granular ICU data enable detailed patient similarity matching and can be used in mortality prediction [16]. Soon, the patient similarity concept will incorporate genomics, proteomics, macrobiotics, and diverse components of system medicines [14]. NetDx [7] uses the data from Cancer Genome Atlas to predict the survival rates across four tumor types, where each tumor type represented a PSN.

### 2.4. Performance Evaluation of the Existing PSNs

The deep patient representation [17] is compared with measures, such as principal component analysis (PCA), K-means, Gaussian mixture model (GMM), and independent component analysis (ICA), using only one transformation with respect to the original data (shallow feature learning). DeepPatient significantly outperformed other feature learning methods, achieving an accuracy of 93%, followed by ICA, K-means, GMM, and PCA with the lowest accuracy being 87.9%. The multidimensional patient similarity [24] supervised approach reported an accuracy of 77%, followed by hierarchical and K-means with 73% and 71%, respectively. The optimized similarity measure [43] with specific term-weighting improved the accuracy (74.3%) associated with diagnosis prediction when compared with equal (73.5%) and generic term-weighting (72.8%) approaches. 

### 2.5. Challenges of the Existing Works

PSN approaches have been recently used in precision medicine, and several challenges must be addressed for them to achieve their full potential. The main challenge is the availability of open datasets. Only a few open datasets exist for patient health data, and most of them require license agreements and extensive deidentification that takes time. Building a representative patient profile is difficult because of the complexities of medical records [8]. Moreover, challenges related to preprocessing, processing, storing, and analyzing big eHealth data in real time from various sources are characterized by its volume and speed. In fact, employing a scalable and distributable scheme, such as MapReduce architecture [44], can address the big data challenge associated with the storage and retrieval of patient data in real time for building PSNs. Data reduction and event sequence summarization from EHR data into features are critical for differentiating between patients [45]. Furthermore, ICD codes, which form the basis of the majority of the PSNs, are often based on a specific country [15]. Another set of key challenges in deriving meaningful PSN measures is how to leverage physician input according to physician feedback, interactively updating the existing similarity measure in real time, and combine different similarity measures from multiple physicians [45]. PSN similarity evaluation is another challenge and would be incomplete without the observational or cumulative aspect of patient resemblance [14]. The prediction performance associated with patient similarity is directly proportional to the degree of similarity between the past and index patient. The converse of this argument is that data from dissimilar patients could degrade predictive performance [16]. Based on the above limitations, our proposed model addresses the following challenges:

(1) Diverse and heterogeneous clinical narrative data enrich hidden information that is valuable in determining the most similar patients. The medical events are temporally sensitive, and the temporal information is critical for comprehending the dynamics of medical terminologies and inferences. The interpretation of temporal representation is extremely difficult when using noisy clinical datasets, and the accuracy of outcome prediction is low.

Our proposed approach addresses both temporal and clinical narrative data by implementing a hybrid model that considers the static and dynamic aspects of patient data in patient similarity analysis that improves accuracy. Static data modeling handles static patient profile data, whereas dynamic data modeling handles longitudinal dynamic data, where each patient is associated with a sequence of visits. Our static model can capture textual unstructured features using Natural Language Processing (NLP) models, such as BERT.

(2) Health datasets exhibit diverse and high dimensionalities. For example, the EHR includes a wide range of information, including diagnosis, medication, laboratory tests, X-rays, and various medical events, such as diseases and medications. Since the data are a mixture of static and dynamic data, accurate modeling and processing are challenging.

Using the generalized hybrid model, the heterogeneity of the eHealth data from various data sources can be managed. Thus, this model is efficient in addressing big data challenges, where the structured and unstructured data of patient cases characterize variety. The reduction in dimensionality is a strategy developed within our model using an autoencoder to achieve a robust and statistically sound machine learning model.

(3) One of the ways to integrate multiple biological data is to concatenate standardized measurements. However, the concatenation of data tends to dilute the data quality with noise. 

Our patient SNF approach utilizes the PSN distance calculations from static and dynamic data that emphasize the similarity of the patient pair and decrease the interference caused by non-similar pairs.

## 3. A Multidimensional Data Fusion Model based on Deep Learning and PSN

In this section, we describe the proposed system architecture in which a DL-based approach was adopted for building patient similarity. We emphasize the main processes involved in implementing our solution, including the data collection phase, DL model development, training, testing, model accuracy evaluation, and diagnostic prediction and clinical recommendations.

### 3.1. Data Collection, Preparation, and Preprocessing

The data of each patient were characterized by demographic and clinical variables, including the recorded vital signs (e.g., blood pressure and heart rate), physical exam findings, symptoms, laboratory tests, and prior medical history. Health data streams were managed using various stream preprocessing approaches, such as PCA, or other data reduction techniques. The processed streamed data were then stored in databases. Various data features can be selected based on the diseases to be predicted. The stored data were queried accordingly and processed to eliminate inconsistent and redundant data. Then, the data were represented in an adequate form accepted by DL algorithms (e.g., vector and matrix).

### 3.2. Architecture: Component Description

**Figure 2:**
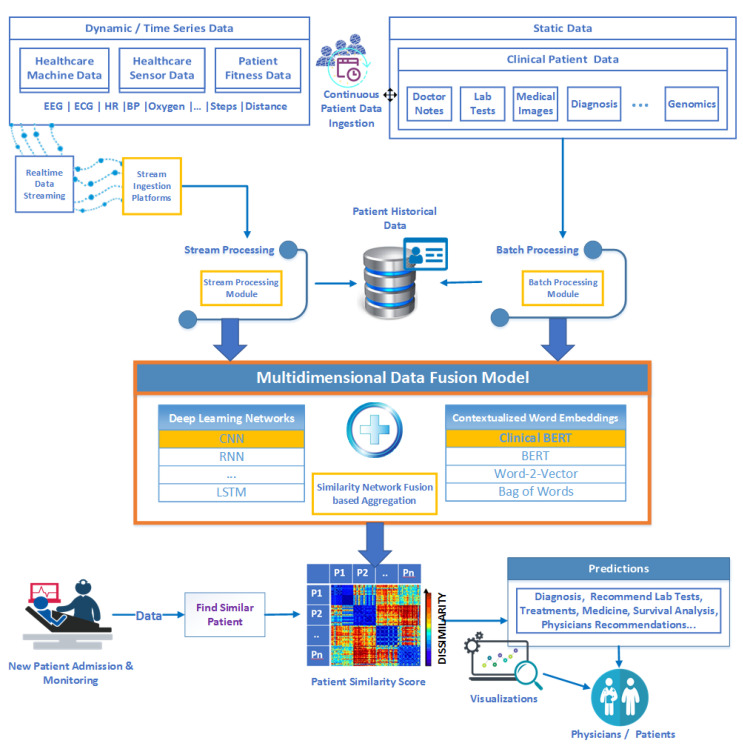
System architecture.

Figure 2 depicts the main components of our system and the key processes involved in data collection, model construction, training, and evaluation. Proactive recommendations will be drawn from the prediction results, which can be obtained from the laboratory test recommendations, medication suggestions, and treatment propositions.

#### 3.2.1. Deep Learning Algorithm Selection

This process involved the exploration of different DL algorithms and selection of the most appropriate algorithm based on various criteria, including the type of machine learning tasks (supervised, unsupervised, semi-supervised, or reinforcement learning), the type of disease to be predicted, the nature of selected features, data size and type (discrete or time-series), and complexity of the model. This selection can be based on the previously conducted studies and a thorough comparison and benchmarking of the different DL models.

#### 3.2.2. Model Development, Training, Prediction, and Evaluation

The model highlights the similarity-network-fusion-based aggregation referred to as the hybrid model (Figure 2). The dynamic data from the stream processing module could benefit the DL model, whereas the clinical static data could employ contextualized word embeddings. The similarity distances were calculated for each patient and combined to output a patient similarity score that serves to find similar patients when a new patient arrives. For prediction model evaluation, we used several performance metrics, including the root-mean-square error (RMSE) and mean absolute percentage error (MAE).

#### 3.2.3. Prediction and Visualization

**Figure 3:**
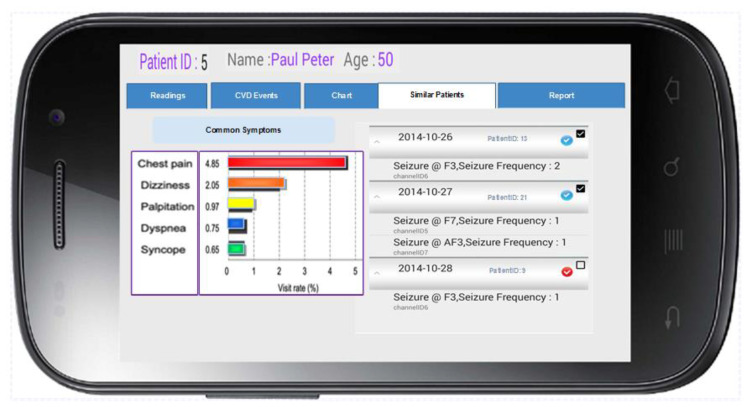
Visualization dashboard—A physician’s perspective.

In this module, a dashboard was designed to visualize the forecast outcomes and collection of guidelines and clinical advice, including diagnosis, potential laboratory examination, and drug prescription. A prototype of the mobile app visualization dashboard, which provides a physician’s perspective of listing similar patients when a specific patient is selected (in this case, patient ID 5), is depicted in Figure 3. It also indicates the common symptoms experienced by similar patients with respect to cardiovascular disease (CVD) events and brain seizures.

### 3.3. Architecture: Technologies, DL Platforms, and Tools

Traditionally in NLP, feature engineering techniques require considerable awareness of the domain and commitment in interpreting meaningful characteristics. The situation is more challenging in the case of the healthcare domain, where clinical machine learning models are difficult to use daily in the case of hospital stays on unstructured, high-dimensional, and fragmented data, such as clinical notes, including laboratory reports, radiology reports, as well as nursing, pharmacy, and physician notes. Reading numerous clinical notes is a tedious task for a physician. However, clinical notes have considerable scientific benefits. Tools that can automate and obtain accurate clinical forecasts are invaluable in medical practice. BERT preprocessing and training are highly computational processes. The authors in [46] proposed a pre-trained fine-tuned BERT model to support researchers’ applications in different domains. Clinical BERT [46,47] is a tool for modeling clinical notes that can discover and allow medical professionals to forecast clinical insights. Similarly, BioBERT [48] is a pre-trained language representation model for the biomedical domain, and biomedical NLP studies may benefit from it. Alsentzer et al. further pre-trained BioBERT on all MIMIC III discharge summaries (DischargeBERT) [46]. BioBERT is the most similar to PubMedBERT [49] since it also pre-trains using PubMed content. However, by completing domain-specific pretraining from scratch, including the use of the PubMed vocabulary, PubMedBERT outperforms BioBERT in most tasks. BlueBERT [50] is a BERT-based model that has been pre-trained on PubMed abstracts and MIMIC III clinical notes. Researchers have come up with an improved procedure for training BERT models, called RoBERTa [51], which includes training the model for longer, with bigger batches, and over more data. Biomedical ALBERT (BioALBERT) [52] is a context-dependent, rapid, and effective language model trained on huge biomedical corpora to overcome the problem of limited training data. BoneBert [53] is a BERT-based labeling system that was trained on a dataset of 6048 X-ray radiology reports and then fine-tuned using a small collection of 4890 expert annotations. Thus, by employing the pre-trained BERT model, features can be mapped into an embedding matrix that serves as input to other classifiers. Further, BERT is proposed as the apt model for static data.

The architecture proposed in this paper (Figure 2) reveals the possibilities of big eHealth data processing technologies represented by stream ingestion platforms as well as stream and batch processing modules. This will respond to the need of handling timely inputs and provide more personalized treatment. Concerning dynamic data, healthcare professionals can utilize a data-driven approach using platforms such as Apache Kafka, a prominent stream ingestion platform, to enable them to ingest real-time health data sources from patients, such as sensors and medical devices.

Data stream processing engines, such as Spark Streaming [54], support native in-memory storage. However, others typically do not provide their own data storage mechanisms, but offer data source and sink connectors to data ingestion mechanisms, such as Kinesis, Kafka, HDFS, and Cassandra. Spark Streaming can be used to collect data streams from live sources and split the data into batches, which are further processed by the Spark engine to produce the final batch. The resulting batches of data from stream processing and the output of the batch processing module using Spark MLib or similar batch processing tools are stored in databases and utilized to train the model. DL networks can use technologies, such as Tensorflow, Keras, PyTorch, and other DL platforms and libraries, for developing the model to calculate patient similarity scores and provide prediction and visualization regarding diagnosis, treatments, and lifestyle recommendations.

The notion of the proposed patient similarity model is a combination or ensemble model, which is multifaceted. Our proposed multidimensional model can be obtained via algorithm aggregation based on SNF in which a DL network and contextual word embeddings of a PSN are combined. Specifically, the patient similarities in clinical diagnosis, imaging, genomics, and time-series data are considered when finding the most similar patient. Hence, the proposed model can efficiently identify similar patients with comorbidities, for example, having multiple medical conditions.

## 4. Model Formulation

We propose a model formulation to represent patients and derive a similarity measure based on the vectors generated from medical events. We extracted a dense and lower-dimensional representation for patients from EHR data, while conserving temporal information.

To model this data, we denoted the patient set as$\;S=\left\{s_{1},\;s_{2},\;\ldots ,\;s_{n}\right\}$, where $s_{i}\;$is the vector of the $i^{th}$ patient and n is the number of patients. This vector comprises a tuple of two main parts, namely, the static part st and dynamic part d, $s_{i}=\left(st_{i},\;d_{i}\right)$. In this section, we describe static and dynamic data modeling, the similarity network, and the PSN construction algorithms.

### 4.1. Static Data Modeling

The static data part St represents the patient’s profile information containing age, gender, multiple laboratory test items, and multiple disease diagnoses. Further, the similarity of a few selected features, such as age, gender, and diabetes, was modeled.

#### 4.1.1. Feature Similarity for Age

We denoted $age_{i}$ and $age_{j}$ as the ages of patients i and j, respectively. We can represent the feature similarity $fs^{1}$ for age as the ratio of the smaller age to the larger age [55].

$$fs_{i,j}^{1}=\frac{\min\left(age_{i},age_{j}\right)}{\max\left(age_{i},age_{j}\right)}$$

#### 4.1.2. Feature Similarity for Gender

For the gender feature, we defined the similarity feature $fs^{2}$ between patients i and j as 1 if they had the same gender and 0 otherwise.

$$fs_{i,j}^{2}=\{\begin{array}{c} 1,\;\;\;\;\;\;\;\;\;\;if\;gen_{i}=gen_{j}\\ 0,\;\;\;\;\;\;\;\;\;\;\;\;\;\;\;\;\;Otherwise \end{array}$$

#### 4.1.3. Feature Similarity for Other Static Features

Other static features included events, such as patients having a chronic disease, represented as a Boolean value. For example, when a patient was diabetic, we defined the similarity feature $fs^{3}$ between patients i and j as 1 if both patients had the same condition (either both diabetic or both nondiabetic) and 0 otherwise.

$$fs_{i,j}^{3}=\{\begin{array}{c} 1,\;\;\;\;\;\;\;\;\;\;if\;diab_{i}=diab_{j}\\ 0,\;\;\;\;\;\;\;\;\;\;\;\;\;\;\;\;\;\;\;\;Otherwise \end{array}$$

#### 4.1.4. Global Static Patient Similarity

We calculated the global patient similarity for static features using the following weighted sum of all the static feature similarities as a single measure of static patient similarity (STPS) for patients i and j. We used a weight vector$\;WV=\left\{w_{1},\;w_{2},\;\ldots ,\;w_{nf}\right\}$, where nf is the number of static features used to evaluate the patient similarity, $w_{k}\;$is the weight given for each static similarity feature$\;fs^{k}$,$\;w_{k}\epsilon \;W$, and$\;\sum_{k=1}^{nf}w_{k}=1$.

$$STPS_{i,j}=\sum_{k=1}^{nf}w_{k}\;fs_{i,j}^{k},\;where\;k=1,\;2,\;3,\ldots ,nf.$$

### 4.2. Dynamic Data Modeling

The dynamic data part D was extracted from the EHR data, which is a time-series vector representing the number of visits m and was denoted by a sequence of visits as$\;D=\{\;PVd_{1},PVd_{2},\;\ldots ,\;PVd_{m}$}. Each visit $PVd_{i}\;$was denoted by a high dimensional vector $\;PVd_{i}$, where each element in that vector ϵ R and indicates that the patient has a medical event value represented as a real number, for example, a patient *p* having a visit $PVd_{i}$, which is a vector containing all medical events that were measured during this visit, such as BMI = 20.80, smoke = 0, diabetic = 0, sbp = 116, dbp = 81, and chol = 214. Therefore, the horizontal axis indicates the rows (i), each of which represents a visit $PVd_{i},\;$and the vertical axis indicates the columns (j), which represent the medical events $x_{i}\;\epsilon \;X$, where X is the set of medical events, that is, features measured during the visit. The ${\left(i,j\right)}^{th}\;$value was observed at time $t_{i}$ of $PVd_{i}\;$for a certain patient. The number of visits varied for different patients. Thus, the dimension of this matrix was defined as $dim=\max{\left(D\right)}_{i=1}^{m}$. This variable-sized data can be managed using an autoencoder-based long short-term memory (LSTM), which is detailed in the following section.

#### 4.2.1. Long Short-Term Memory (LSTM)

LSTM [56] is a variation of deep RNNs that have been commonly adopted in diverse domains, such as language modeling and speech recognition. A typical LSTM network is comprised of different memory blocks called cells. There are two states that are being transferred to the next cell: the cell state and the hidden state. The memory blocks are responsible for remembering things and manipulations to this memory are achieved through three major mechanisms, called gates (Forget Gate, Input Gate, and Output Gate). LSTM quickly learns to differentiate between two or more widely spaced instances of a given element in a series of inputs. Learning rate, input gate bias, and output gate bias are just a few of the factors that LSTM excels at. RNN is designed for sequential data, such as time-series, text, audio, and video data. Contrary to a standard feedforward neural network, RNN considers the input data at the current time step and the output of the previous time step [57]. In addition, RNN involves cycles with network activations from a previous time step as inputs to the network, affecting the predictions at the current time step, and incorporates the memory of previous events. Nevertheless, standard RNN exhibits issues, such as vanishing and exploding gradients, which affect long-term dependencies [58]. LSTM overcomes vanishing gradient problems using a forget gate that allows the error to be backpropagated through time and via layers, allowing gradients to flow unaffected through many time steps [59]. 

Choosing LSTM in our autoencoder model facilitated the feature reduction process to learn from the temporal relationships among time-series features, instead of implementing a feature reduction process that flattened all the time-series features and lost the temporal information contained in the set of features. We first trained our dataset utilizing a reconstruction autoencoder model to reduce the size from 20,680 to 4046 rows with 5D embeddings each. Choosing 5D embeddings produced good accuracy results when training. The proposed model used a batch of series of patient exam records as input and output (1 × 5) vectors that is the final hidden state. We used the rectified linear activation function (ReLU) in our LSTM model and the loss values were calculated based on the mean square error (MSE). In our model, LSTM was a gated RNN with an input vector, which is the dynamic part vector $d_{i}\epsilon \;R^{S}$ of the patient’s set PV.

#### 4.2.2. Patient Visit Matrix Embedding (Data Dimension Reduction)

The dynamic data part D was fed into one layer of the time-series LSTM model encoder to preserve the temporal features of patients’ data. This layer reduced the data dimension to produce an output vector$\;D^{\prime }$, which included embeddings of a smaller dimension d as the final hidden state. This was performed to reduce data dimensions and learn relationships among features. Thus, each column was embedded in the vector space. Consequently, each visit $dPV_{i}$ was mapped into an embedding matrix $EB_{i}\epsilon \;R^{dim}$, where dim<|X| the embedding dimension. Using the rectified linear activation function (ReLU), the summed weighted input was transformed into an output using a formula similar to that in a previous study [12], where $W_{v}\epsilon \;R^{dim*\left|X\right|}\;\mathrm{and}\;b_{v}\epsilon \;R^{dim}$ are the weight matrix and bias vector to be learned, respectively.

$$e_{i}=ReLU\;\left(W_{v}\;V_{i}+b_{v}\right)$$

ReLU(x)=max(0,x)

This operation resulted in an embedding matrix $EB_{i}\;$for each patient, resulting in a lower feature dimension than that of the original dataset.

### 4.3. Similarity Network Fusion

SNF is a new nonlinear computational approach for integrating and fusing different PSNs [18]. It combines different datasets. In our study, the static and dynamic similarity matrices were aggregated for a given dataset of patients, achieving good performance. This approach begins with the construction of a sample similarity network for each data matrix. In this work, we used the static data matrix STM and the dynamic data matrix DM, which were formed using algorithms 1 and 2, respectively, depicted in Section 5. Then, we iteratively integrated such networks using a network fusion method described as follows. First, we normalized each matrix by dividing each row element of the matrix by the sum of the rows, so that the sum of all the elements in each row was 1.

$$w_{i,j}=\frac{m_{i,j}}{\sum_{j=1}^{n}m_{i,j}},$$

where $w_{i,j}$ is the normalized value of each element $m_{i,j}\;$of the similarity matrix. Then, the normalized matrix W can be symmetrized as

$$W_{Sym}=\left(W+W^{T}\right)/2\;,\;$$

where $W^{T}\;$denotes the transpose of W. The resulting matrices were defined as STM  and DM to represent the static data similarity matrix and dynamic data similarity matrix, respectively.

Next, we used the K-nearest neighbor method to calculate the local similarity for each matrix [18].

$$w_{i,j}^{\prime }=\{\begin{array}{c} \frac{w_{i,j}}{\sum_{y\;\epsilon \;W}w_{i,j}^{y}},\;\;j\;\epsilon \;N\\ 0,\;\;Otherwise \end{array},$$

where N is a set of nearest neighbors of patient i from both matrices denoted by y with size K determined by the user. Thus, the strongest links with the highest weights were selected, and the weak links in the network were eliminated to reduce noise interference. Finally, the two updated matrices $ST{M^{\prime }}^{1}\;$ and $D{M^{\prime }}^{2}$, formed by calculating the local similarity using the above equation, were fed to the SNF algorithm that iterated for a given number of iterations T, starting at $MP_{t=0}^{1}=STM\;$ and $\;MP_{t=0}^{2}=DM$. In general, SNF fuses the similarity networks attained from different data types separately by aggregating their data. The resultant fused network captures the integrated information obtained from different data sources, that is, by fusing the similarity between all patients rather than a pair of patients. However, in this paper, we used SNF to combine patient similarity matrices rather than raw data. Therefore, we modified the algorithm to aggregate the similarity values between each pair of patients into a single value in accordance with the following aggregation function based on the weighted average [60].

(1) $$MP_{t+1}^{1}=(wt_{s}\;STM^{\prime }+\left(1-wt_{s}\right)\;MP_{t}^{2})\;/2,\;MP_{t+1}^{2}=(wt_{d}\;DM^{\prime }+\left(1-wt_{d}\right)\;MP_{t}^{1})\;/2,$$

where $wt_{s}\;\mathrm{and}\;wt_{d}$ denote the weights according to the significance of each matrix estimated by the user. Here, $MP_{t+1}^{1}$ is the state matrix transformed based on the STM similarity matrix after t iterations and $MP_{t+1}^{2}$ is the state matrix transformed based on the DM similarity matrix after t iterations. In each iteration, the information of each similarity network was changed to produce two final state matrices that were integrated into the fusion similarity matrix FM as:

$$FM=\left(MP_{t}^{1}+MP_{t}^{2}\right)/2,where\;t=T$$

**Figure 4:**
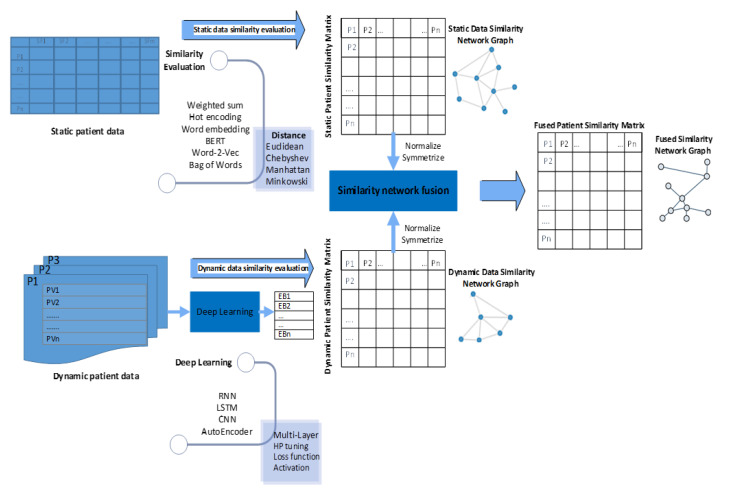
Key processes in building a PSN.

This modification distinctly indicated the strength of similarity between each pair of patients and reduced the noise and interference that can be attributed to the similarity of other patients. This integrated matrix, which was obtained from the sequential operations, produced a PSN defined as a graph G = (V, E). The vertex V represents the patient set S, and the edges E are weighted by the similarity level between the patients. The edge weights were denoted as a N×N similarity matrix FM resulted from the final iteration of the SNF algorithm, as explained earlier, where each element $w_{i,j}$ indicates the similarity level between patients $s_{i}$ and$\;s_{j}$. Figure 4 shows the key processes associated with the building of a hybrid PSN, including static, dynamic, and fused similarity matrix constructions, as per the formal description.

## 5. PSN Construction Algorithms

In this section, we describe our algorithms for constructing the proposed hybrid PSN. We developed three algorithms. The first algorithm implements the procedure to generate the static similarity matrix, the second algorithm implements dynamic similarity matrix generation, and the third algorithm implements similarity matrix fusion.

Algorithm 1 outputs the STPS matrix based on the model explained in this study. The input to this algorithm is the static data part of the patient dataset, the list of selected features to be evaluated for similarity, the list of similarity utility for each selected feature, and the given weights for each similarity feature.

**Algorithm 1.** Static data similarity evaluation algorithm 
**Input:**
*PList*,*▷* List of Patients *SFList*,*▷* List of selected features *SUList*,*▷* List of similarity utility for each feature 
*weights*
*▷* List of weight for each feature 
**Output:**

*SSM*
*▷* Static similarity matrix for all patients  1: **procedure** STATICSIMILARITYMATRIX(*PList*, *SFList*, *SUList*, *weights*)  2:  *SSM* ← *initilizeToEmpty*() 3:  **for** *s_i_* ← 1*,N* do           *▷* each patient i    4:    **for** *s_j_* ← *s_i_* + 1*,N* do       *▷* each patient j  5:     **for** *f_k_* ← 1*,K* do      *▷* each selected feature (col)  6:        *FSscore*[*s_i_,s_j_*] ← *getSimilarityScore*(*s_i_,s_j_,SUList*[*f_k_*]) 7:        *SSM*[*s_i_,s_j_*] ← *SSM*[*s_i_,s_j_*]+*FSscore*[*s_i_,s_j_*]∗*weights*[*f_k_*] 8:     **end for** 9:    **end for** 10: **end for** 11: **return** *SSM* 12: **end procedure** = 0

Algorithm 2 applies the DL autoencoder model to generate the dynamic similarity matrix. It takes as input the dynamic data part of the patient list denoted as PV, the activation function, e.g., ReLu, the number of dynamic features to be evaluated, and the output embedding dimension.

**Algorithm 2.** Dynamic data similarity evaluation algorithm
**Input:**
*DPList*,*▷* List of Patients with dynamic data *ACTF*,*▷* Activation function *NF*,*▷* Number of features 
*NEMB*
*▷* Embedding dimension
**Output:**

*DSM*
*▷* Dynamic similarity matrix for all patients  1: **procedure** DYNAMICSIMILARITYMATRIX(*DPList*, *ACTF*, *NF*, *NEMB*) 2: *preprocess*(*DPList*)  3: *EB* ← *deepLearningAutoencoder*(*DPList,ACTF,NF,NEMB*) 4: **for** *s_i_* ← 1*,N* do              *▷* each patient i  5:   **for** *s_j_* ← *s_i_* +1*,N* do           *▷* each patient j 6:     *DSM*[*s_i_,s_j_*] ← *getSimilarityScore*(*EB*[*s_i_*]*,EB*[*s_j_*]) *▷*Euclidean 7:   **end for** 8: **end for** 9: **return** *DSM* 10: **end procedure**=0

Algorithm 3 finalizes the fusion process. It takes as input the two matrices, the number of nearest neighbors K, and the number of iterations T required for executing the iterative SNF process. The final output is the fused patient matrix referred to in this study.

**Algorithm 3.** Similarity network fusion algorithm
**Input:**
*STM*,*▷* Static similarity matrix *DM*,*▷* Dynamic similarity matrix *T*,*▷* Number of iterations to complete fusion *K*,*▷* Number of nearest neighbors *wt_s_*,*▷* Weight for Static similarity matrix 
*wt_d_*
*▷* Weight for Dynamic similarity matrix 
**Output:**

*FPSM*
*▷* Fused patient similarity matrix  1: **procedure** SIMILARITYNETWORKFUSION(*STM*,*DM*,*T*,*K*) 2: *M*^1^*_prev_* ← *STM* 3: *M*^2^*_prev_* ← *DM* 4: *normalize*(*STM,DM*) 5: *symmetrize*(*STM,DM*) 6: **for** *s_i_* ∈ *STM*
**do**       *▷* calculate local similarity for STM 7:  *neighborList*←*nearestKNeihbors*(*s_i_,k,STM,DM*) 8:  **for** *s_j_* ∈ *neighborList*
**do** 9:    
$STM\left[si,sj\right]\leftarrow STM\left[si,sj\right]/\sum_{i=1}^{k}neighborList\left[i\right]$
 10:  **end for** 11: 
**end for**
 12: **for** *s_i_* ∈ *DM*
**do**        *▷* calculate local similarity for DM 13:  *neighborList* ← *nearestKNeihbors*(*s_i_,k,STM,DM*) 14:  **for** *s_j_* ∈ *neighborList*
**do** 15:   
$DM\left[si,sj\right]\leftarrow DM\left[si,sj\right]/\sum_{i=1}^{k}neighborList\left[i\right]$
 16:  
**end for**
 17: 
**end for**
 18: **for** *t_i_* ← 1*,*
*T*
**do** 19:  
$M^{1}\;\leftarrow \;\left(wt_{s}\times STM+\left(1-wt_{s}\;\right)\times M_{prev}^{2}\right)/2$
 20:  
$M^{2}\;\leftarrow \;\left(wt_{d}\times DM+\left(1-wt_{d}\;\right)\times M_{prev}^{1}\right)/2$
 21:  
$M_{prev}^{1}\;\leftarrow \;M^{1}$
 22:  
$M_{prev}^{2}\;\leftarrow \;M^{2}$
 23: 
**end for**
 24: *FPSM* ← *FM* = (*M*^1^ +*M*^2^)*/*2 25: **return** *FPSM* 26: **end procedure** = 0

## 6. Experimentation and Result Discussion

In this section, we describe the experimental setup and tools, dataset, and details of the experiments, after which the obtained results will be discussed.

### 6.1. Experimental Setup

For our experiments, we used Google Colab notebooks, with DL framework Tensorflow, machine learning packages from Scikit-learn, SciPy, and BERT with Configuration {“attention_probs_dropout_prob”: 0.1, “hidden_act”: “gelu”, “hidden_dropout_prob”: 0.1, “hidden_size”: 768, “initializer_range”: 0.02, “intermediate_size”: 3072, “max_position_embeddings”: 512, “num_attention_heads”: 12, “num_hidden_layers”: 12, “type_vocab_size”: 2, “vocab_size”: 28,996}, which is a transformer-based machine learning technique for NLP pretraining for our batch processing. We also developed an autoencoder-based DL module and performed PSN distance computation (Figure 4). Further, we implemented the PSN construction, including the static, dynamic, and fusion matrix construction algorithms previously explained in this study, and performed a matrix performance evaluation using JAVA via Apache NetBeans IDE version 12.2 from the Apache Software Foundation. 

### 6.2. Dataset

We used two data sources throughout our experiments. (1) Dataset-1 was the epidemiological COVID-19 data [61], which were compiled and assembled from the state, regional, and local health reports. The data are geocoded and contain symptoms, primary dates (date of onset, admission, and confirmation), chronic diseases, travel history, and admission status for multiple COVID-19 patients. We used the data collected until 30 August 2020, including 155 complete records after preprocessing and cleaning, each of which represents an individual patient case. The dataset has 33 columns with four class outcomes (death, discharged, stable, and recovered). This dataset was selected for experimenting with the clinical text data and primarily includes symptoms, chronic disease, and additional information; NLP can be applied in this case. (2) Dataset-2 was the Framingham offspring heart study [62], which is a long-term cardiovascular cohort study including adult offspring of the original Framingham study that began in 1949 (Framingham, MA, USA). A total of 5124 individuals were recruited from 1971 to 1975 and were followed up for many years to examine secular trends in cardiovascular disease and its risk factors and also to investigate the association between risk factors and the incidence of cardiovascular disease, including stroke, myocardial infraction and CVD death. Details about the Framingham offspring cohort (https://biolincc.nhlbi.gov/studies/framoffspring/ (accessed on 1 March 2022)) utilized in the research and information about all Framingham cohort studies (https://biolincc.nhlbi.nih.gov/studies/fhs/ (accessed on 1 March 2022)) are available.

We adopted this dataset in our experiment because it considers the dynamicity of patient data characteristics. Further, multiple visiting records and static features were considered for each patient to evaluate our proposed fusion algorithm. 

Table 2 summarizes the principal features of the two datasets used in our experiments.

### 6.3. Evaluation Criteria

In our experiments, we compared the different distance algorithms used to generate our proposed similarity matrices and selected the optimal similarity matrix. Furthermore, we compared the performance of the fused matrix with the performance of the static and dynamic data similarity matrices independently. We evaluated the similarity matrices by adopting different evaluation criteria, such as accuracy, precision, recall, and F1-score [63]. We summarized our similarity matrix model evaluation using a 2 × 2 confusion matrix that depicted all four possible outcomes: true positive (*TP*), false positive (*FP*), false negative (*FN*), and true negative (*TN*).

*TP*: accurate prediction of similar patients (predicted that two patients are similar and both died).

*TN*: accurate prediction of non-similar patients (predicted that two patients are not similar, and both have different outcomes., e.g., P1 died and P2 survived).

*FN*: similar patients inaccurately predicted as non-similar (predicted two patients as non-similar, but they both have similar outcomes).

*FP*: non-similar patients inaccurately predicted as similar patients (predicted two patients as similar, but they have different outcomes).

We adopted the following measurements to validate and compare the performances of our similarity matrices as follows.

$$Accuracy=\frac{TP+TN}{TP+TN+FP+FN}=\frac{correctly\;predicted\;similar\;and\;non-similar\;patients}{total\;number\;of\;predictions}$$

$$Recall=\frac{TP}{TP+FN}=\frac{correctly\;predicted\;similar\;patients}{correctly\;predicted\;similar\;patients+similar\;patients\;incorrectly\;predicted\;as\;non-similar}$$

$$Precision=\frac{TP}{TP+FP}=\frac{correctly\;predicted\;similar\;patients}{correctly\;predicted\;similar\;patients+non-similar\;patients\;incorrectly\;predicted\;as\;similar}$$

$$Precision=\frac{TP}{TP+FP}=\frac{correctly\;predicted\;similar\;patients}{correctly\;predicted\;similar\;patients+non-similar\;patients\;incorrectly\;predicted\;as\;similar}$$

$$F1\;Score=\frac{2*\left(Recall\;*\;Precision\right)}{Recall+Precision}$$

### 6.4. Experimental Scenarios

We conducted a series of experiments to evaluate our proposed multidimensional PSN using two different datasets. We adopted two principal experimental scenarios. In the first scenario, we focused on evaluating the similarity matrices generated based on a mixture of numerical and textual clinical data. In the second scenario, we focused on the performance of the SNF model that aggregates the static and dynamic features of patient data. Throughout all our experiments, we compared the performance of different geometrical distance algorithms, including Euclidean, Manhattan, cosine, Chebyshev, and weighted Manhattan, for patient similarity calculations. The goal of any machine learning project is to construct a more generic model that can perform well on unknown data, thus we chose k-fold cross-validation [64], one of the most popular strategies extensively utilized by data scientists. The fivefold cross validation approach was used in our experiments, which divided the training dataset into five parts, each of which having been chosen as the validation dataset for testing. The accuracy of the experiments was evaluated based on the equation in Section 6.3. Our experimental scenarios were aligned to validate the following objectives.

1. Scenario 1 evaluated the PSN model, where the data exhibited static features with a mixture of numerical and textual data.
  - ICU admission prediction for COVID-19 patients based on Dataset-1.
  - Evaluate the accuracy of the patient similarity matrix while using NLP models, BERT, and one-hot-encoding. These models were adopted to better capture the semantics of the clinical textual data and find the most similar patient.
  - Identify the best similarity distance measurement approach among the Euclidean, Manhattan, cosine, Chebyshev, and weighted Manhattan approaches.
  - Determine the optimal weight distribution among features when using the weighted distance evaluation approach. This approach improves accuracy when giving more significance to certain features than others.
  - Evaluating the PSN model performance when applying the local similarity approach for the similarity matrix can limit data conflicts and improve accuracy.
2. Scenario 2 evaluated the overall performance of our proposed multidimensional model, where the dataset involved a combination of dynamic and static features.
  - Predict a CVD event in the future based on Dataset-2.
  - Build a static PSN matrix for the static portion of the data and evaluate the performance of the STPS matrix according to the evaluation criteria mentioned in this study.
  - Evaluate the performance of the autoencoder used for the dynamic portion of the patient data for data reduction, thereby compacting the input patient information into a lower-dimensional space.
  - Build and evaluate the performance of the dynamic similarity matrix.
  - Evaluate the performance of the fused similarity matrix based on our proposed SNF algorithm and confirm that our model can represent the large, heterogeneous, and dynamic contents of a dataset.

#### 6.4.1. Scenario 1. PSN Evaluation on Static Data having Numerical and Textual Data 

Dataset-1 was used for this scenario, wherein both numerical and clinical textual data were available. The effectiveness of the static algorithm solution and distance estimation were evaluated. Further, the classification performance was analyzed using a fivefold cross-validation method. The accuracy, recall, precision, and F1-score measures were calculated, as explained in the evaluation criteria of this study, to compare the performances of different similarity distance calculation algorithms.

1. Accuracy measure of patient similarity

**Figure 5:**
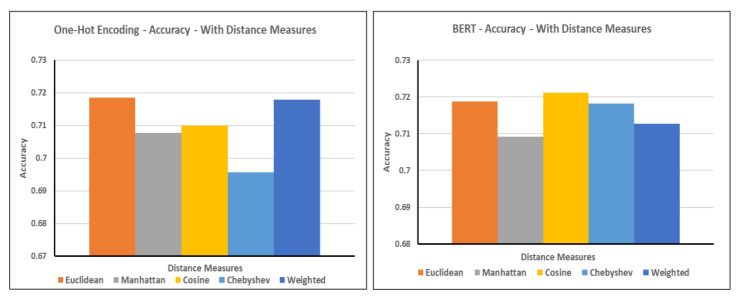
Accuracy with various distance measures (one-hot encoding and BERT).

In this scenario, we generated numerical representations from the contextual embedding of textual clinical data via hot encoding and BERT. Next, we evaluated the accuracy of the resulting patient similarity matrix using different distance calculation techniques, including Euclidean, Manhattan, cosine, Chebyshev, and weighted approaches (Figure 5). The graphs illustrate that the Euclidean and weighted distance calculations performed better in accuracy for one-hot encoding, whereas cosine excelled when using BERT.

Table 3 presents the results obtained based on the performance evaluation parameters of various distance measures used in one-hot encoding and BERT. The overall performance of BERT is slightly better than that of one-hot encoding.

2. Weighted-Distance Accuracy Measure against Similar Patients

In this experiment, we evaluated the patient similarity matrix generated using the weighted Manhattan distance algorithm after BERT contextual encoding. We defined different weights for each feature to provide more significance to some features over others that were validated based on medical expertise.

We employed a weighted scoring approach [65], a prioritization framework to prioritize the features and determine the weights for the current scenario. The set of weights were given to the six features, namely, age, gender, symptoms, additional_information, chronic_disease_binary, and chronic_disease, as shown in Table 4. We assigned various weights to each feature to give certain features more importance than others, which was confirmed by medical experts.

Then, we assigned scores for each feature option ranging from 1 to 4. The default weight was 1. We used the following guidelines to assign weight scores:

- To boost the score contribution, we set the weight to higher than 1.
- To maintain the score contribution, we set the weight to 1.

**Figure 6:**
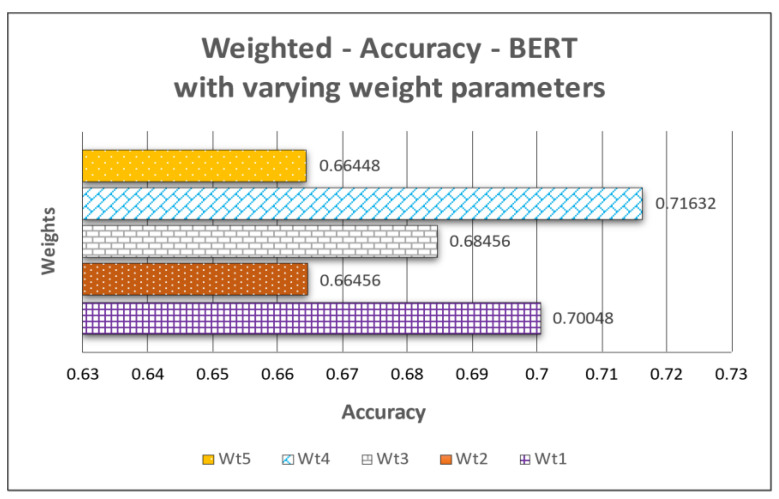
Weighted accuracy based on weighted features.

Wt1 = [1,1,3,3,3,1], Wt2 = [1,1,3,2,3,3], Wt3 = [1,1,4,3,2,2], Wt4 = [1,1,3,3,3,3], and Wt5 = [1,1,3,1,1,1] represent the sets of weights assigned to age, gender, symptoms, additional_information, chronic_disease_binary, and chronic_disease, respectively. Optimal results were obtained when the features (symptoms, additional_information, chronic_disease_binary, and chronic_disease) of Wt4 were assigned higher weights, as represented (Figure 6).

3. Accuracy Measure against the Selected Percentage of Similar Patients

Our next step in the experiment was based on the strategy of using the K-nearest neighbors of similar patients to calculate the local similarity for each matrix to increase the prediction accuracy. The details of this approach are depicted in this study.

**Figure 7:**
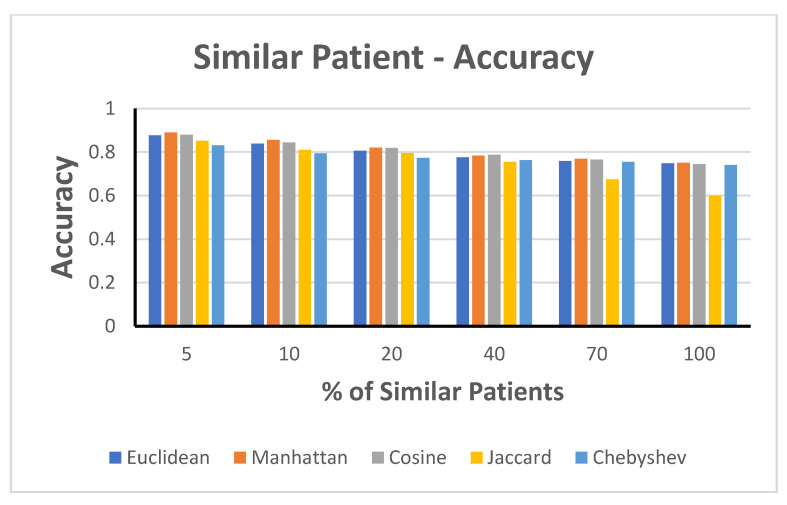
Accuracy with varying training data involving similar patients.

The results presented (Figure 7) show that improved outcome prediction results can be obtained by considering only similar patients. The highest accuracy of 89% could be obtained for the Manhattan approach when selecting 5% of the related patients in our training, whereas selecting the full data (100%) resulted in a mere 75% accuracy. Thus, selecting the optimum number of similar patients was crucial to improve the predictive performance and decrease the training time (a key factor when big health data are considered).

#### 6.4.2. Scenario 2. Hybrid PSN Model Evaluation Data with Static and Dynamic Features 

In this scenario, we adopted Dataset-2, which is a combination of patient static demographic and dynamic longitudinal data, indicating multiple patient visits, which is ideal for evaluating our proposed fusion model. The class attribute in this dataset was developing CVD.

1. Static PSN Evaluation

**Figure 8:**
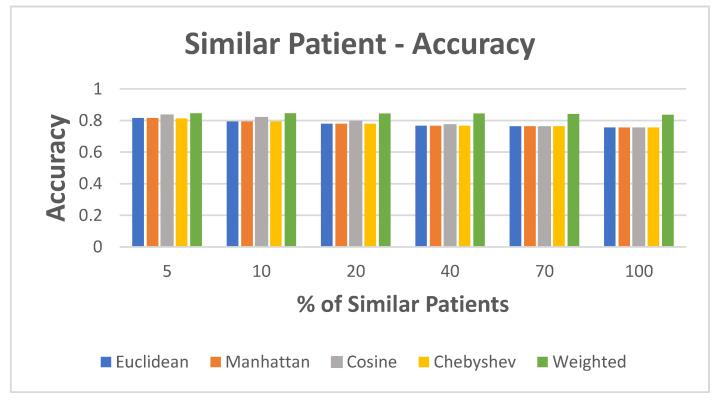
Static data: accuracy in the case of similar patients.

In this experiment, we evaluated the accuracy of the STPS matrix based on different distance calculation algorithms. Table 2 presents the static features used for similarity. We evaluated the accuracy based on the different K-nearest neighbor values of similar patients. Accuracy increased when closely similar patients were selected for training the model, that is, the K-value decreased, as depicted in Figure 8. The weighted distance measurement resulted in the highest accuracy, at 83–84%, among all trials, followed by the cosine distance measure with an accuracy of 83% when considering 5% similar patients and 75% when using the full dataset. All the remaining distance measures resulted in improved results when the training data included the most similar patients.

2. Dynamic PSN Evaluation (Autoencoder)

**Figure 9:**
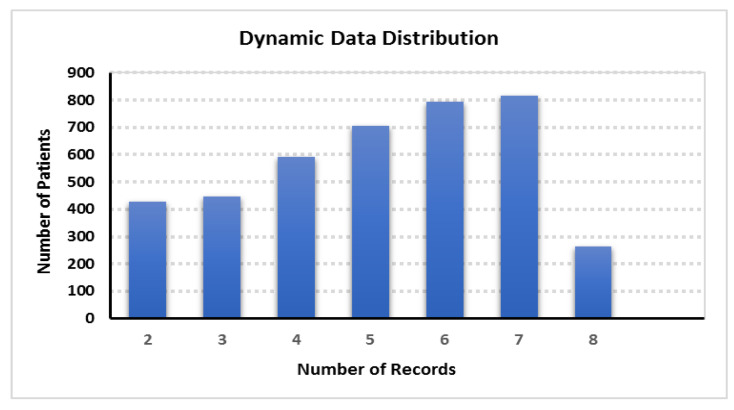
Dataset 2: dynamic data distribution.

In Dataset-2 (CVD dataset), each patient has a different number of records representing the health measurements associated with each visit, which dictates reducing data dimensionality to facilitate the construction of the dynamic PSN. We first trained our dataset utilizing a reconstruction autoencoder model to reduce the size from 20,680 to 4046 rows with 5D, 32D, and 64D embeddings each. Subsequently, we trained the autoencoder model-generated output into a similarity matrix using one of the different distance measurement approaches. First, we split our dataset into static profile data and dynamic time-series patient visit records. Figure 9 presents the dynamic data balanced distribution, for example, approximately 400 patients have 2 records each and 800 patients have 7 records each.

We made a random search to fine-tune the hyperparameters of our autoencoder. We followed a simple algorithm to train the model with the hyperparameters, chosen by intuition and experience, and then tried different combinations of hyperparameter values using cross-validation and measured the MSE to decide on the optimal combination of values for the hyperparameters.

For the embedding dimensions, we compared the accuracy using values ranging from 5 to 64. This experiment depicted a better accuracy for dimension 32. Accordingly, we decided to use an embedding dimension value of 32, which increased the accuracy of the fused matrix and gave us better overall results. Similarly, we compared the MSE when using a different number of layers, ranging from 1 to 3. The results show that using one hidden layer worked well with our problem, although a slight improvement was achieved when using a higher number of layers, which did not justify the extra time spent for training. In other words, more layers can be better, but also harder to train, so we decided to choose one layer for faster training.

**Figure 10:**
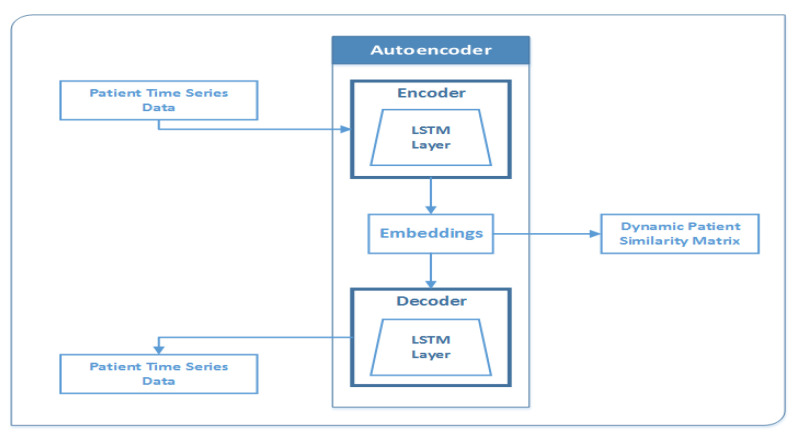
The architecture of the data reduction autoencoder.

**Figure 11:**
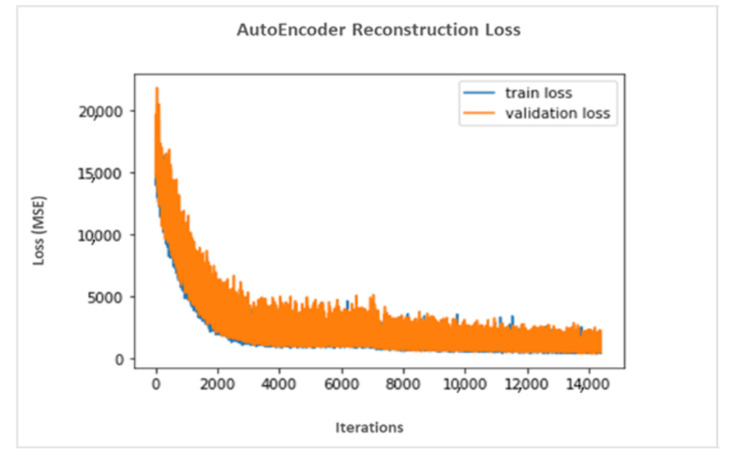
Reconstruction loss associated with an autoencoder.

In summary, the dynamic part of our data was fed into an LSTM layer. The proposed model used a batch of series of patient exam records as input and output (1 × 32) vector that is the final hidden state. However, the decoder used the (1 × 32) vector and passed it to an LSTM layer, which produced the dynamic time-series part. Figure 10 describes the architecture of the LSTM-based encoder–decoder neural network developed for data reduction. The following are the parameters used for the $LSTM\;\left(input_{size},\;hidden_{size},\;num_{layers}\right)$, where the $input_{size}$ is the number of expected features (x = 9), the $hidden_{size}$ is the number of features in the hidden state h = 32, and $num_{layers}$ is the number of recurrent layers = 1. Additionally, the set of inputs were $\left(input,\;\left(h_{0},\;c_{0}\right)\right)$, where the input is a tensor of shape $\left(batch_{size},\;sequence_{length},\;input_{size}\right)$ having a $batch_{size}$ of 32, a $sequence_{length}$ that is a variable depending on the number of rows (visits) for an individual patient, and an $input_{size}$ = 9, i.e., the number of features. In our experiment, $h_{0}$ was a tensor of shape $\left(num_{layers},\;batch_{size},\;H_{out}\right)$, where $num_{layers}$ = 1, $batch_{size}$ = 32, and $H_{out}$ = 32. Furthermore, $c_{0}$ is a tensor of shape $\left(num_{layers},\;batch_{size},\;hidden_{size}\right)$, where $num_{layers}$ = 1, $batch_{size}$ = 32, and $hidden_{size}$ = 32. Figure 11 illustrates the autoencoder reconstruction loss values obtained based on MSE while generating (1 × 32) vector embedding. In this model, the reconstruction loss values decreased gradually and stabilized after approximately 3000 iterations.

3. Fusion PSN Evaluation

**Figure 12:**
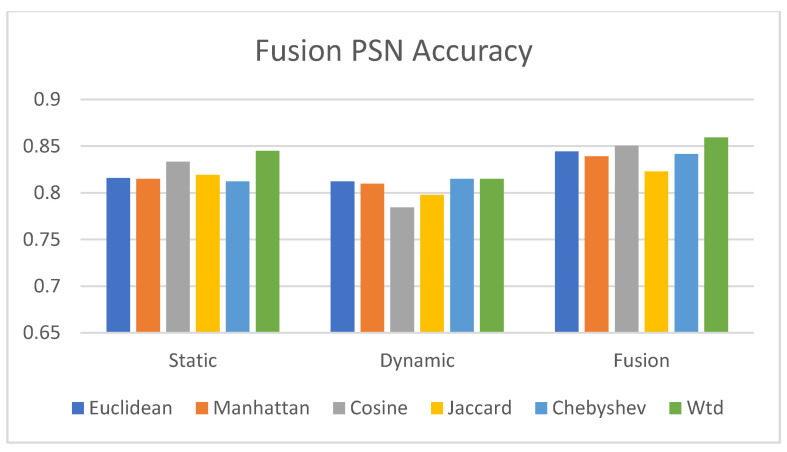
Accuracy of the fusion PSN.

In this experiment, we evaluated the performance of the resultant fused patient similarity matrix against the outcome class with respect to the different distance measurements explained in this study. Figure 12 depicts the performance of the final PSN matrix when compared with the static and dynamic similarity matrices while adopting different distance measurements. Our proposed SNF approach improved the accuracy of the final fusion patient similarity matrix when compared with the accuracies of the static and dynamic similarity matrices.

Our experimental evaluation (Figure 12) also discloses that the static PSN data provided more accuracy than the dynamic PSN data. Here, the dataset consisted of static data, such as gender, age, and diabetic status, which featured categorical values with little variance. However, the dynamic features included frequently changing time-variant fields, such as BMI, Chol, and LDL, and each patient had a varying number of hospital visits (Figure 9). According to our view, the variance in static and dynamic data components, as well as the differences in PSN calculation methods, such as data reduction using autoencoders in dynamic PSN calculation, resulted in a considerable difference in accuracy. Similar studies depicted that autoencoders may cause accuracy reduction [66]. Another study on the performance of autoencoder with Bi-Directional LSTM [67] reported that the accuracy and F1-score of the model with an autoencoder dropped by around 4% and 9%, respectively, indicating that some information is lost because the encoding process does not hold all of the information from the original data. Moreover, as per Chen [68], even if the epoch size is high, the accuracy will be less than the initial accuracy because encoding and decoding cause some data loss. We believe this holds true in our above experiment using autoencoder for data reduction as well, where accuracy variation is around 1–5% between the static and dynamic PSN data.

#### 6.4.3. Scenario 3. Benchmark to Other Classification Algorithms

Our multi-model PSN can be used for unsupervised or supervised data with high accuracy. To validate this, we selected one of the features as a labeled outcome to convert unsupervised learning into a supervised learning technique. Further, we evaluated the similarity network matrices with respect to this outcome. The experimental results show that a higher accuracy is achieved by the fused similarity matrix when compared with those of both the static and dynamic data similarity matrices when evaluated independently.

Furthermore, we benchmarked our PSN model with other widely adopted classification algorithms using the CVD and COVID-19 datasets. Accuracy improvement can be obtained by performing classification using our multi-model PSN when compared with those of other baseline-supervised classification models, such as Logistic Regression, Naïve Bayes, ZeroR, Decision Tree, and Random Forest. The parameters used in the chosen classification models were:

- **Naïve Bayes**: {var_smoothing = 1e-09}
- **SVM:** {‘SVMType’: C-SVC, ‘KernelType’: 2, ‘Degree’: 3, ‘nu’: 0.5, ‘cachesize’: 40, ‘cost’: 1, ‘eps’: 0.001, ‘loss’:0.1}
- **ZeroR**: {‘batchsize’: 100, ‘useKernelEstimator’: False, ‘useSupervisedDiscretization’: False}
- **CNN**: {‘layer’: 5, ‘Out’: 2, ‘gradNormThreshold’: 1.0-minimize, ‘algorithm’: STOCHASTIC_GRADIENT_DESCENT, ‘updater’: Adam, ‘biasUpdater’: Sgd, ‘weightInit’: XAVIER, ‘learningRate’: 0.001, ‘numEpochs’: 10 “}
- **Logistic Regression**: {‘C’: 1.0, ‘dual’: False, ‘fit_intercept’: True, ‘intercept_scaling’: 1, ‘max_iter’: 100, ‘multi_class’: ‘auto’, ‘penalty’: ‘l2′, ‘solver’: ‘lbfgs’, ‘tol’: 0.0001, ‘warm_start’: False}
- **RandomTree:** {‘KValue’:0, ‘minNum’: 1, ‘minVarianceProp’:0.001, ‘seed’: 1}
- **Decision Tree:** {‘ccp_alpha’: 0.0, ‘criterion’: ‘gini’, ‘min_samples_leaf’: 1, ‘min_samples_split’: 2, ‘splitter’: ‘best’}

Table 5 presents the different accuracy results of the classification algorithms. When testing using the CVD dataset results, the accuracy improved by 20% when compared with that of naïve Bayes; further, a minimum of 10% improvement could be observed when compared with those of zeroR and decision tree. However, experiments on the COVID-19 dataset show that our model results in a 7% higher accuracy than those of zeroR and LR and around 1–3% improvement compared with the other models. We included a CNN model that was the second best in accuracy for the CVD Dataset, scoring 91.2%, indicating that our proposed PSN model outperforms the neural network models as well.

## 7. Conclusions

Although data-driven prediction in personalized medicine is a developing field, the data analytics paradigm has been successfully applied in other research fields, such as personalized product recommendation in e-commerce. PSN is a new model to integrate data to cluster patients, and it has exciting potential for personalizing and improving healthcare. Although several data mining and DL models have been used to build PSNs and apply them, a single model cannot cope with the heterogeneity of the data and their large dimensionality, while maintaining a high accuracy and preserving the veracity of the data. Therefore, in this study, we proposed a multidimensional model that captures both contextual and longitudinal data and addresses the data dimensionality problem. In this model, DL models were combined with PSNs to provide richer clinical evidence and extract relevant information based on which similar patients can be compared and explored. BERT was used for contextual data analysis and the generation of embeddings, whereas CNN was used to capture the semantic features. In addition, an LSTM-based autoencoder was developed for data dimensionality reduction while preserving temporal features. A fusion model was developed to aggregate the results obtained from the two models and proposed more precise diagnoses and recommendations for a new patient. A set of experiments was conducted to evaluate the accuracy of our DL-based PSN fusion model. The results proved that the model provides a higher classification accuracy in determining various patient health outcomes when compared with other traditional classification algorithms.

Five potential directions are available for further improvement: (1) establish how PSN can be applied in survival analysis and implement a cardiovascular risk calculator; (2) address scalability issues when similarity matrices increase in size; (3) enhance the model to support values other than classes of nominal outcomes; and (4) improve the model with thorough experiments because the methodology is a new (5) experiment with a few of the BERT model variations described in Section 3.2, such as BioBERT, Dis-chargeBERT, PubMedBERT, BlueBERT, RoBERTa, and BioALBERT.

The PSN paradigm, for example, can be used to improve patient outcomes, provide treatment or drug recommendations to new patients, predict clinical outcomes, and provide clinical decision support. The trust associated with the recommendations can be considerably improved using new and continuously added data. Network-based patient similarity approaches have conceptual and technical features that are crucial to enable precision medicine.

## Figures and Tables

**Table 1:** Methods used for building patient similarity.

Method	Parameters/Factors	Applications
Deep learning	ICD9	Unsupervised/supervised patient similarity (CNN) [8] Diagnosis with LSTM recurrent neural networks [11] Personalized disease prediction (CNN) [10]
Triplet-loss metric learning	Longitudinal EHRs	Personalized prediction [12]
Temporal similarity	Temporal sequences	Clinical (workflow) case similarity [13]
Clustering	Variety of components of patient data	Patient similarity analytics loop [14]
Similarity measure construction	ICD code, Empirical co-occurrence frequency, Medical history, Blood test, ECG, Age, Gender	Predict individual discharge diagnoses [15] Predict ICU mortality [16]
Deep patient representation (three-layer stacked denoising autoencoders)	ICD9	Future disease prediction [17]
Similarity network fusion (SNF)	Nodes represent patients, and patients’ pairwise similarities are represented by edges	Network-based survival risk prediction Identifying cancer subtypes [18]
Locally supervised metric learning (LSML)	Longitudinal patient data	Personalized predictive models and generation of personalized risk factor profiles [19]
Collaborative filtering methodology	ICD data	Creates a personalized disease risk profile and a disease management plan for the patient [20]
Anonymous indexing of health conditions for a similarity measure	Text similarity	Recommend two other patients for each patient based on a keyword [21]
SimSVM	14 similarity measures from relevant clinical and imaging data	Predicting the survival of patients suffering from hepatocellular carcinoma (HCC) [22]
Concept hierarchy	Hierarchical distance measure	Detecting correlations in medical records by comparing the hierarchy of terms considering the distance between non-similar records in a hierarchy [23]

**Table 2:** Summary of the datasets used in our experiments.

	Dataset-1	Dataset-2
**Dataset Based On**	COVID-19	CVD
**Type**	Static	Static and Dynamic
**Size**	Small (200)	Big (20,000)
**Fields**	**Static**: ID, age, gender, date_onset_symptoms, date_admission_hospital, date_confirmation, symptoms, additional_information, chronic_disease_binary, chronic_disease, outcome	**Static**: PID, exam_age, gender, smoke, diab, hypermed, age_baseline, smoke_baseline, gender_baseline, diab_baseline, hypermed_baseline, time_long_years, time_to_event_years **Dynamic**: Bmi, sbp, dbp, chol, hdl, ldl, trig non_hdl, chol_hdl_ratio, time_long_years, time_to_event_years, time_long_scal, time_to_event_scal

**Table 3:** Evaluation of the PSN distance measures with one-hot encoding and BERT.

		One-Hot Encoding				BERT		
	Accuracy	Accuracy Std. Dev.	Precision	F1-Score	Accuracy	Accuracy Std. Dev.	Precision	F1-Score
**Euclidean**	71.86	4.78	72.10	83.35	72.37	4.77	99.73	83.73
**Manhattan**	70.78	5.63	71.01	82.62	72.28	5.52	99.89	83.70
**Cosine**	71.00	5.24	71.24	82.68	84.60	5.51	97.64	89.97
**Chebyshev**	69.58	5.70	71.90	80.98	72.12	5.61	99.66	83.59
**Weighted**	71.79	5.40	72.33	82.82	71.83	4.99	96.93	83.04

**Table 4:** Weighted scoring table.

	Age	Sex	Symptoms	Addnl_Info	Chronic_Disease_Binary	Chronic_Disease	Score	Rank
Weight	0.1	0.15	0.2	0.15	0.2	0.2		
Option1	1	1	3	3	3	1	**2.1**	**4**
Option2	1	1	3	2	3	3	**2.35**	**2**
Option3	1	1	4	3	2	2	**2.3**	**3**
Option4	1	1	3	3	3	3	**2.5**	**1**
Option5	1	1	3	1	1	1	**1.4**	**5**
Option6	2	1	1	2	1	1	**1.25**	**9**
Option7	1	1	1	1	1	1	**1**	**10**
Option8	1	2	2	1	1	1	**1.35**	**6**
Option9	1	1	1	2	1	2	**1.35**	**7**
Option10	1	1	1	2	2	1	**1.35**	**8**

**Table 5:** Benchmark PSN model compared to other classification algorithms.

Dataset	Accuracy							
	PSN	Naïve Bayes	SVM	ZeroR	CNN	Logistic Regression	Random Tree	Decision Tree
CVD Dataset 2	96%	80.67%	87.20	87.03%	91.2%	87.10%	87.32%	87.03%
COVID-19 Dataset 1	89%	84.80%	88.45	83.20%	85.84%	83.20%	88.80%	86.40%

## Data Availability

Not applicable.
